# A Role for Na^+^,K^+^-ATPase α1 in Regulating Rab27a Localisation on Melanosomes

**DOI:** 10.1371/journal.pone.0102851

**Published:** 2014-07-22

**Authors:** Antonia E. G. Booth, Abul K. Tarafder, Alistair N. Hume, Chiara Recchi, Miguel C. Seabra

**Affiliations:** 1 Molecular Medicine, National Heart and Lung Institute, Sir Alexander Fleming Building, Imperial College London, London, United Kingdom; 2 School of Biomedical Sciences, University of Nottingham, Medical School, Queens Medical Centre, Nottingham, United Kingdom; 3 CEDOC, Faculdade de Ciencias Medicas, FCM, Universidade Nova de Lisboa, Lisboa, Portugal; University of Tennessee, United States of America

## Abstract

The mechanism(s) by which Rab GTPases are specifically recruited to distinct intracellular membranes remains elusive. Here we used Rab27a localisation onto melanosomes as a model to investigate Rab targeting. We identified the α1 subunit of Na^+^,K^+^-ATPase (ATP1a1) as a novel Rab27a interacting protein in melanocytes and showed that this interaction is direct with the intracellular M4M5 loop of ATP1a1 and independent of nucleotide bound status of the Rab. Knockdown studies in melanocytes revealed that ATP1a1 plays an essential role in Rab27a-dependent melanosome transport. Specifically, expression of ATP1a1, like the Rab27a GDP/GTP exchange factor (Rab3GEP), is essential for targeting and activation of Rab27a to melanosomes. Finally, we showed that the ability of Rab27a mutants to target to melanosomes correlates with the efficiency of their interaction with ATP1a1. Altogether these studies point to a new role for ATP1a1 as a regulator of Rab27a targeting and activation.

## Introduction

Rab GTPases are essential regulators of intracellular membrane trafficking [1]. Key to this activity is their ability to associate with specific membrane compartments within the cell [2]. Although much progress has been made in assigning functions to individual Rabs and identifying their downstream effectors, the mechanism(s) by which precise Rab targeting is achieved remains elusive [3]. A number of mechanisms have been postulated including the involvement of the C-terminal hypervariable domain [4]–[7], Rab effector binding [8], GDI displacement factors [9]–[11] and RabGEFs [12]–[15], all of which has been summarised in a recent review [16].

Rab27a regulates the transport and secretion of lysosome-related organelles and secretory granules from a variety of cell types including melanocytes, haematopoietic cells (T-lymphocytes, mast cells, natural killer cells and neutrophils), endothelial cells, platelets and neuroendocrine cells [17], [18]. In melanocytes, Rab27a associates with melanosomes and recruits its effector Melanophilin (Mlph/Slac2-a/Exophilin 5), which binds the actin-based motor protein MyosinVa (MyoVa). The formation of the Rab27a∶Mlph∶MyoVa tripartite complex is essential for the peripheral distribution of melanosomes and efficient melanosome transfer to neighbouring keratinocytes [18]–[22]. Loss of any member of the tripartite complex results in perinuclear clustering of melanosomes [22]–[24]. For further details on melanogenesis the author directs the reader to the following reviews [25]–[27].

Previous work in our laboratory has investigated the involvement of the aforementioned mechanisms in the targeting of Rab27a to melanosomes in melanocytes. We demonstrated that exchange of the C-terminal hypervariable domain of Rab27a with Rab1a or Rab5a did not re-target the hybrid protein and similarly, the Rab27a C-terminal domain was insufficient to retarget Rab5a or Rab1a [28]. Furthermore, exchange of the Rab Family (RabF) and Rab SubFamily (RabSF) motifs [29] demonstrated that elements of the RabSF2 and RabSF3 motifs were crucial for Rab27a targeting to melanosomes [28]. Importantly, the Rab27a mutant containing elements of the RabSF2 motif of Rab5 (Rab27a^SF2^) maintained interactions with known Rab27a effectors yet failed to target to melanosomes [13], thus indicating that effector binding is not sufficient for Rab27a targeting. This was supported by the observation that a Rab27a mutant with the RabSF1 and RabF4 motifs of Rab3 (Rab27a^SF1/F4^) failed to bind to any known Rab27a effector yet maintained its melanosomal localisation showing that effector binding is not necessary for targeting [13]. Interestingly, depletion of Rab3GEP (R3G), the non-redundant Rab27a GEF in melanocytes [30], was able to disrupt Rab27a targeting [13] suggesting a key role for GEF activity. This is consistent with the results of a number of recent studies showing that GEF activity is essential for targeting of Rabs to specific membranes [3], [15], [31], [32]. However, the Rab27a^SF2^ mutant was found to be efficiently GTP-loaded by R3G *in vitro* yet mis-targeted in melanocytes, therefore indicating that the GEF activity alone is not sufficient for Rab27a targeting to melanosomes in melanocytes [13]. More recently, work in yeast showed that a Ypt7 mutant with improved nucleotide exchange activity was still correctly localised in the absence of the GEF, thereby supporting the concept that in addition to the GEFs, other factors are required for Rab targeting [15]. Taken together, we hypothesised that in combination with the role of R3G, Rab27a may interact with additional putative ‘targeting factor(s)’ that contribute to the recruitment of Rab27a to melanosomal membranes. In this study we aimed to identify such targeting factor(s) for Rab27a using a tandem affinity purification strategy. Our results indicate the α-subunit of the Na^+^,K^+^-ATPase isoform 1 (ATP1a1) as a key Rab27a targeting factor in melanocytes.

## Materials and Methods

### Antibodies

For Western blotting the following antibodies were used at the concentrations indicated: mouse anti-Rab27a, 1∶10000 [22]; rabbit anti R3G, 1∶5000 (a kind gift of J. Myoshi and Y. Takai); mouse anti-GST, 1∶10000 (Sigma G1160); mouse anti-tubulin, 1∶5000 (Sigma T6557); mouse anti-ATP1a1, 1∶5000 (Abcam ab7671); rabbit anti-His, 1∶1000 (Abcam ab9108); mouse anti Pmel17, 1∶500 (Dako M0634); goat anti-GPNMB, 1∶1000 (R&D Systems AF2330); rabbit anti-Annexin A2, 1∶5000 (a kind gift from J. Ayala-SanMartin CNRS/UPMN/ENS).

For immunofluorescence the following antibodies were used at the concentrations indicated: rabbit anti-Rab27a, 1∶200 (purified by R. Singh, Imperial College, UK); mouse anti-ATP1a1, 1∶200 (Thermo MA1-16731); rabbit anti-Rab5a, 1∶50 (Abcam ab18211); rabbit anti-Rab38, 1∶100 (purified by C. Wasmeier, Imperial College, UK [33]); rabbit anti-Mlph (purified by A. Hume, Imperial College, UK [34])

For immunoprecipitation the monoclonal anti-HA (Roche 12CA5) and anti-FLAG (Sigma F3165) were used.

### siRNA Oligonucleotides

All siRNA oligonucleotides were purchased from Dharmacon (Thermo Scientific). Non-targeting (NT) ON-TARGETplus (D-001810-02) was used as a negative control. siRNA oligos for Mlph and R3G were detailed previously [13]. On-TARGETplus SMARTpools for ATP1a1 (L-052829-00), Pmel17 (L-063274-01), GPNMB (L-040019-01), and Anxa2 (L-061993-01) and individual ON-TARGETplus ATP1A1 oligos were purchased and optimal ATP1a1 depletion was obtained using oligo 5′-GUAUGAGCCUGCCGCUGUA-3′ (J-052829-06).

### Plasmid Constructs

HTF-Rab27a constructs were generated by PCR amplification of rat Rab27a^WT^ and Rab27a^SF1/F4^ from pEGFP-C2 vectors described previously [13] using primers designed to incorporate 5′ NheI and 3′ SacI restriction sites. The PCR products were sub-cloned into a cPSG5PL vector encoding a Hemagglutinin-TEV-FLAG (HTF)-tag and Hygromycin-B resistance gene (a gift from S. Baron; Stanford University) resulting in N-terminally HTF-tagged Rab27a. GFP-tagged ORF cDNA for human ATP1a1 was purchased from OriGENE. GST-ATP1a1-M4M5 was generated by PCR amplification of a 1269 bp fragment of hATP1a1 (Lys354–Lys777) encoding the M4M5 loop. Primers were engineered to introduce 5′ EcoRI and 3′ SalI restriction sites. The PCR product was sub-cloned into a pGEX-4T1 vector.

### Cell Culture

All tissue culture material was purchased from Invitrogen unless otherwise specified. Mouse WT melanocytes (melan-INK4a) and melan-ink4a-*ashen* (melan-*ash2*) [35] were maintained in RPMI 1640 (+L-Glutamine) supplemented with 10% fetal calf serum, 100 U/mL penicillin, 100 U/mL streptomycin, 200 nM Phorbol-1,2-myristate-1,3-acetate (PMA; Calbiochem) and 200 pM Cholera Toxin (CT; Sigma) (Complete RPMI medium). All cells were cultured at 37°C under a humidified atmosphere containing 10% CO_2_. Cells and melanosome distribution was assessed using a light microscope. For immunofluorescence experiments, cells were grown on glass coverslips in 24-well plates. Transfection of plasmid DNA was performed using FuGENE6 (Roche Diagnostics) in a 1∶3 ratio of µg of DNA to µl of FuGENE6, according to the manufacturer's recommendations. For each well of a 24-well plate, 0.5 µg of DNA was mixed with 1.5 µl of FuGENE6 in 25 µl of serum-free medium OptiMEM. Following a 5 h incubation, the medium was replaced with RPMI (+L-Glutamine) supplemented with 10% (v/v) FBS. After overnight culture the medium was replaced with complete RPMI medium. For siRNA analyses, cells were transfected with siRNA to a final concentration of 100 nM using Oligofectamine (Invitrogen) according to the manufacturer's instructions. Following 5 h incubation the medium was removed and replaced with complete RPMI medium. For optimal siRNA depletion the siRNA treatment was sometimes repeated after 72 h. For membrane/cytosol fractionation experiments soluble (S100) and insoluble (P100) fractions were separated by centrifugation at 100'000×g of the PNS. The P100 was then resuspended in the same volume of loading buffer as the total volume of S100. Equal volumes of S100 and P100, relative to the protein concentration of the PNS for each siRNA treatment, were loaded on the gel. Quantification of ECL signals was carried out using ImageJ software.

### Generation of Stable Mouse Melanocyte Cell Lines

Transfection of melan-*ash2* was carried out as described above. Two days post transfection, the medium was replaced with complete RPMI medium supplemented with 100 µg/ml Hygromycin B (Invitrogen). Cells were grown under selection for three weeks before Hygromycin-resistant cells were sub-cultured to one cell per well in a 96-well plate. The isolated clones were expanded and sub-cultured to increasing culture volumes.

### Tandem Affinity Purification

Three 150 cm^2^ flasks of stable HTF-Rab27a expressing melan-*ash2* cells were harvested by trypsinisation, washed in PBS and the pellets flash frozen in liquid nitrogen. Cell pellets were resuspended in 300 µl Buffer A (50 mM Tris pH 7.5, 150 mM NaCl, 5 mM MgCl_2_, 1 mM DTT and protease inhibitor cocktail (PI) (Roche)) and lysed by passage through a 25G needle. CHAPS was added to a final concentration of 1% (Buffer A^+CHAPS^) and the sample incubated for 1 h at 4°C, rotating. The soluble fraction (S100) was isolated by centrifugation at 100,000×*g* for 1 h at 4°C. The S100 was pre-cleared by incubation with 100 µl pre-equilibrated Protein A/G Ultralink Resin (beads; Thermo Scientific) for 1 h at 4°C, rotating. 50 µg of mouse anti-HA antibody was added to the pre-cleared S100 and incubated for 2 h at 4°C, rotating. This was added to 250 µl pre-equilibrated beads and incubated for 2 h at 4°C, rotating. The beads were washed 3 times in Buffer A^+CHAPS^ and resuspended in 200 µl Buffer A^+CHAPS^ including 10 µl (10 U) AcTEV Protease (Invitrogen) and incubated overnight at 4°C, rotating. The supernatant was isolated and incubated with 13.6 µg of mouse anti-FLAG antibody for 2 h at 4°C, rotating. This was added to 50 µl pre-equilibrated beads and incubated for 2 h at 4°C, rotating. The beads were washed 3 times in Buffer A^+CHAPS^ and resuspended in 100 µl Buffer A^+CHAPS^ including 45 µg of FLAG peptide (Sigma) and incubated for 2 h at 4°C, rotating. The eluted sample was then isolated. Multiple TAPs were performed with lysates of HTF-Rab27a^WT^ and HTF-Rab27a^SF1/F4^ expressing melan-*ash2* cell lines and untransfected melan-*ash2* cells; the samples were pooled and sent for Mass Spectrometry analysis at Dundee Cell Products.

### Silver Staining of SDS Polyacrylamide Gels

The SDS polyacrylamide gel was fixed in Fixer (50% MeOH, 12% Acetic Acid, and 0.05% Formalin) for 2 h at room temperature. The gel was then washed 3 times for 20 min in 35% EtOH before sensitising in 0.02% Na_2_S_2_O_3_ for 2 min, then washing 3 times for 5 min in H_2_O. The gel was stained in Silver Nitrate (0.2% AgNO_3_, 0.076% Formalin) and washed twice for 1 min in H_2_O before adding to Developer (6% Na_2_CO_3_, 0.05% Formalin, and 0.0004% Na_2_S_2_O_3_). Developing was stopped by transferring to Stop Solution (50% MeOH, 12% Acetic Acid) for 5 min.

### Recombinant Proteins

Recombinant GST-tagged ATP1a1-M4M5 was expressed in *Escherichia coli* BL21-codon plus (DE3) RILP (Stratagene) and purified using a method adapted from [36]. In brief, 600 ml Luria Broth was inoculated with an overnight culture to obtain A_600_ = 0.1 and grown for 3 h until A_600_ = 0.6. Isopropyl β-D-1-thiogalactopyranoside (VWR) was added to a final concentration of 0.1 mM and the culture grown for an additional 4 h. The bacterial pellet was washed in PBS then stored at −20°C before resuspension in lysis buffer (50 mM Tris pH 7.5, 150 mM NaCl, 5 mM MgCl_2_, 1 mM DTT, 1 mg/ml Lysozyme and PI). This was incubated on ice for 30 min before sonication. The soluble proteins were separated from cell debris by centrifugation 12,000×*g* for 10 min. The bacterial lysate was incubated with 1.5 ml Glutathione Sepharose (GE Healthcare) for 2 h at 4°C, rotating. The Sepharose was washed with Buffer A before eluting GST-ATP1a1-M4M5 with elution buffer (50 mM Tris pH 7.5, 500 mM NaCl, 5 mM MgCl_2_, 1 mM DTT, 10 mM Glutathione). Eluted fractions containing protein as assessed using Bradford Reagent (Pierce), were dialysed (Snakeskin Pleated Dialysis Tubing, 10,000 MWCO, Thermo) in 1000× volume of wash buffer overnight and the buffer replaced for an additional 4 h dialysis. GST and GST-Slp1 [30] and his_6_-tagged Rab27a, -Rab5a and -Rab27a mutant variants [37] were as described previously.

### GST Pulldown

5×10^6^ melan-INK4a cells per condition were resuspended in 500 µl Buffer A and lysed by passing 20 times through a 25G needle. The lysate was cleared by centrifugation at 700×*g* for 5 min at 4°C. CHAPS was added to a final concentration of 1% and incubated for 30 min at 4°C, rotating. The soluble fraction was isolated by centrifugation at 100,000×*g* for 40 min at 4°C. 400 pmol of GST or GST-ATP1a1-M4M5 was added to the soluble fraction with 50 µl equilibrated glutathione-Sepharose and incubated overnight at 4°C, rotating. The Sepharose was washed 5 times with 1 ml Buffer A^+CHAPS^. Bound proteins were eluted by boiling the Sepharose in SDS loading buffer, and the proteins were separated by SDS-PAGE and analysed by immunoblotting using antibodies specific for Rab27a and GST.

The *in vitro* GST pull-down method was adapted from [38]. Briefly, 100 pmol his_6_-Rab was mixed with either 100 pmol GST or GST-ATP1a1-M4M5 in Buffer A^+CHAPS^ and incubated for 30 min at RT, rotating. Equilibrated glutathione-Sepharose (20 µl) was added to each reaction and incubated for 30 min at room temperature, rotating. The Sepharose was pelleted by centrifugation and washed five times with 1 ml Buffer A^+CHAPS^. Bound proteins were eluted by boiling the Sepharose in SDS loading buffer, and the proteins were separated by SDS-PAGE and analysed by immunoblotting using antibodies specific for Rab27a, GST or His. Nucleotide loading of Rab27a was as described previously [39]. Briefly, 100-µl aliquots of Ni^2+^-agarose beads were incubated with 2 nmol of his_6_-Rab27a in Buffer A for 20 min at room temperature. The beads were then washed twice with Buffer A before the bound nucleotide was eluted by washing with 1 M Guanidine-HCl, and followed by two washes with ice-cold Buffer A. The immobilized his_6_-Rab was nucleotide loaded by incubation for 10 min on ice in 200 µl of Buffer A supplemented with 200 µM of either GTPγS or GDP. The beads were then washed rapidly, three times with Buffer A, before the his_6_-Rab was eluted from the Ni^2+^-agarose beads by incubating for 15 min at room temperature with 2 bed volumes of Elution Buffer (600 mM imidazole, 400 mM NaCl). The eluted Rab was then diluted 4-fold in Buffer A^+CHAPS^ and 0.5 mM GTPγS or GDP, and incubated with GST fusion proteins in the presence of 20 µl of glutathione-Sepharose beads for 20 min at room temperature, rotating. The beads were then washed 4 times in Buffer A^+CHAPS^ containing the appropriate nucleotide. Bound proteins were eluted by boiling the Sepharose in SDS loading buffer, and the proteins were separated by SDS-PAGE and analysed by immunoblotting using antibodies specific for Rab27a and GST.

### Effector Pulldown Assay

Melan-INK4a cells were plated in a 6-well plate and treated with NT, ATP1a1 and/or R3G siRNA. Following siRNA depletion, the cells were washed in PBS then 150 µl Buffer A was added. Cells were harvested by scraping and lysed by passage through a 25 G needle. PNS was isolated by centrifugation at 800×*g* for 10 min at 4°C. 25 µg total protein PNS, 20 µl pre-equilibrated glutathione-Sepharose and 20 µM GST or GST-Slp1 were combined. Samples were incubated for 45 min at room temperature, rotating. Sepharose was washed three times with Buffer A. Bound proteins were eluted by boiling the Sepharose in SDS loading buffer, and the proteins were separated by SDS-PAGE and analysed by immunoblotting using antibodies specific for Rab27a and GST. Band intensities were measured using Image J. Rab27a band intensities were normalised to GST-Slp band intensities and then measured relative to NT siRNA control. The experiment was repeated five times and the mean values relative to NT controls presented with standard errors of the mean.

### Immunofluorescence Microscopy

Cells plated on coverslips were washed in PBS and fixed with 4% (w/v) paraformaldehyde (PFA) in PBS for 20 min. They were washed twice in PBS and once with 50 mM NH_4_Cl in PBS, and then permeabilised in PBS containing 2% FBS and 0.05% Saponin (PFS). Permeabilised cells were incubated with primary antibody diluted in PFS, washed three times in PFS and incubated with the appropriate fluorescently-conjugated secondary antibody (Molecular Probes) diluted 1∶400 in PFS. The coverslips were washed three times in PFS, twice in PBS and once in H_2_O, then mounted on microscope slides with either ImmunO-fluore mounting medium (MP Biomedicals) or Prolong Gold antifade reagent (Invitrogen). Microscopy was performed in the Facility for Imaging Light Microscopy (FILM) at Imperial College London. Immunofluorescence images were acquired at room temperature on a Zeiss AxioVert 200M laser scanning inverted confocal microscope ((LSM)-510) using Plan-Apochromat 63× 1.40 Oil DiC M27 objective, Zeiss AxioCam HRm camera and LSM 510 acquisition software. Images were processed with LSM Image Browser and Adobe Photoshop 8.0 software before compiling in Adobe Illustrator.

### Melanosome Purification

Melanosome purification was adapted from [40]. siRNA treated Melan-INK4a were grown until confluent in a 150 cm^2^ flask and then harvested by trypsinisation. Washed cells were then resuspended in 5 ml of chilled Homogenisation Buffer (HB) (250 mM Sucrose, 50 mM Imidazole, 1 mM EDTA, 10 mM MgCl_2_, 0.15 mg/ml Casein, 1 mM DTT and PI) and, lysed on ice using a cell cracker. PNS was then collected by centrifugation at 600× *g* for 5 min at 4°C. The PNS was then centrifuged 3×10 min at 2500× *g* at 4°C and the pellets collected and pooled (P2500). The P2500 was resuspended in 2 ml of chilled HB and 500 µl aliquots layered on top of 500 µl 50% Percoll in microcentrifuge tubes. The sample was then centrifuged 20 min at 8000× *g* at 4°C. The pellets were collected, pooled and diluted up to 1.5 ml HB and centrifuged 10 min at 5000× *g* at 4°C. The final pellet was resuspended in 200 µl HB.

For immunofluorescence analysis, the purified melanosomes were diluted 1∶500 in HB then 500 µl aliquoted onto coverslips in a 24-well plate. The plate was centrifuged 1500× *g* for 5 min at 4°C. Coverslips were fixed in 4% PFA and immunostained as described above.

Immunofluorescence images were taken on a fluorescent confocal microscope as described above. The images were then analysed using Simple PCI Software (Hamamatsu) to measure the average fluorescence intensity of each melanosome. For each experiment five images of melanosomes was taken. The mean fluorescence intensity of the melanosomes in a field of view (60×) was measured and then the average from five images was normalised to experimental NT siRNA controls. The experiment was repeated five times and the mean of the five experiments presented with standard error of the mean.

## Results

### Purification of novel Rab27a interacting proteins from melanocytes

**Figure 1:**
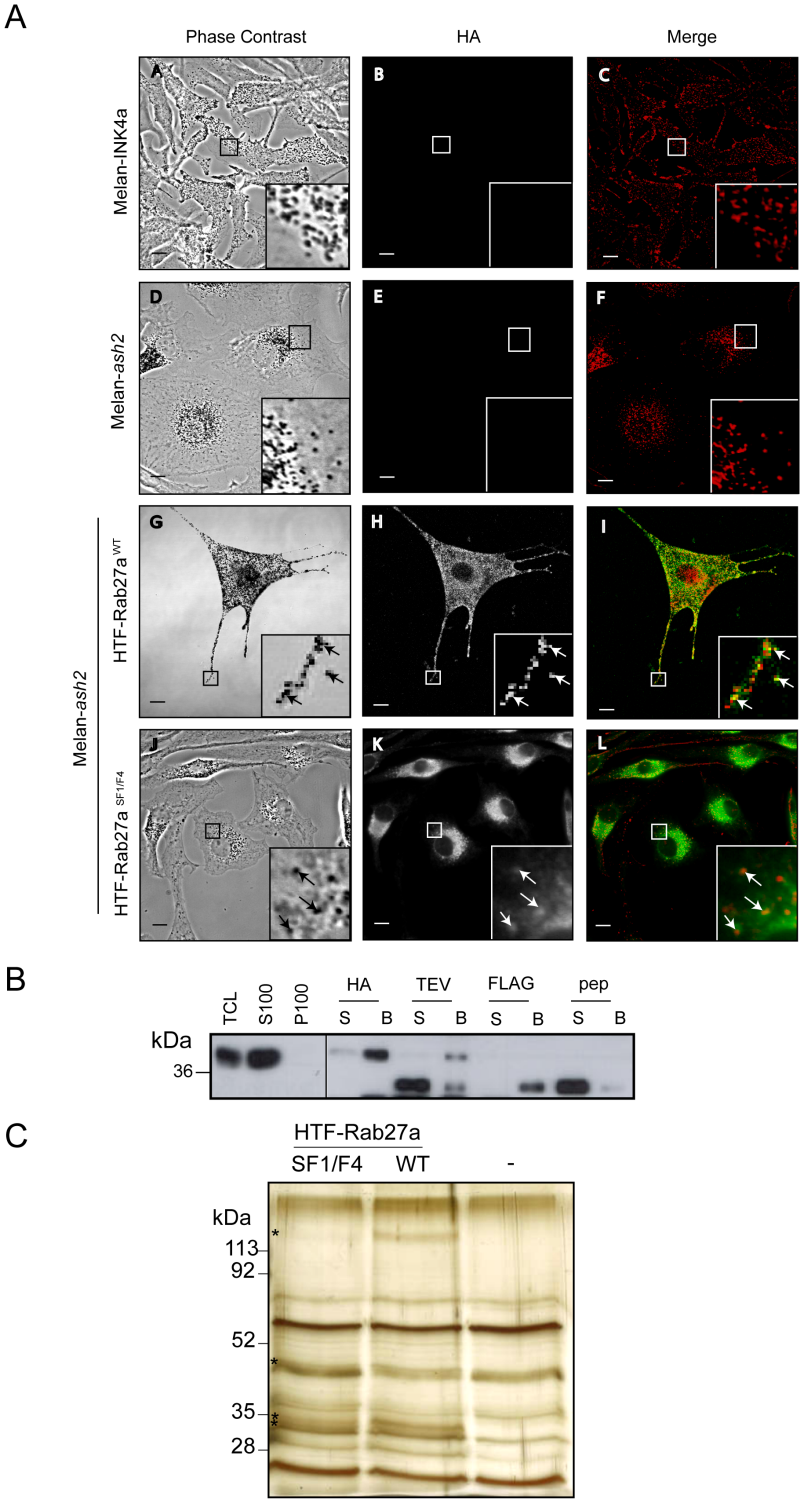
Generation of melan-*ash2* cell lines stably expressing HTF-Rab27a^WT^ or HTF-Rab27a^SF1/F4^ and their use for TAP. Melan-*ash2* cells were stably transfected with HTF-Rab27a for TAP. A) The cells indicated were grown on coverslips and fixed with 4% PFA. Cells were permeabilised and blocked with FBS before immunostaining with mouse anti-HA antibody and Alexa-488 conjugated anti-mouse secondary antibody. Phase contrast panels (A, D, G, J) show melanosome distribution. Panels B, E, H and K show HA immunostaining. Pigment is inverted and pseudo-coloured red to aid colocalisation with HA (green) in the merge panels (C, F, I, L). Insets are a higher magnification of the boxed area. Arrows indicate colocalisation. Scale bar represents 10 µm. B,C) HTF-Rab27a^WT^ or HTF-Rab27a^SF1/F4^ and associated proteins were purified from stably transfected Melan-*ash2* cells by solubilising in 1% CHAPS followed by TAP involving HA immunoprecipitation, TEV cleavage, FLAG immunoprecipitation and FLAG peptide elution steps. B) Immunoblotting for Rab27a of equivalent volumes of each stage of the TAP shows the purification of HTF-Rab27a. C) Silver stained gel shows the final eluted samples (50 µg) of the pooled HTF-Rab27a^WT^, HTF-Rab27a^SF1/F4^ and untransfected Melan-*ash2* TAPs. S100, soluble fraction; P100, pellet fraction; HA, hemagglutinin; TEV, TEV Protease; pep, FLAG peptide. * indicates possible Rab27a interacting proteins.

To identify protein(s) involved in targeting of Rab27a to melanosomal membranes, a tandem affinity purification (TAP) of epitope tagged Rab27a from melanocytes was performed. To this end, Rab27a null melanocytes (melan-*ash2*) were transfected with vectors allowing stable expression of tagged Rab27a (HTF-Rab27a; see Material and Methods), either wild-type or the Rab27a^SF1/F4^ mutant. The latter was used as it targets to melanosomes, but does not interact with known Rab27a/b effectors e.g. Slp/Slac and Munc13-4 proteins [13], and thus would increase the likelihood of identifying novel Rab27a binding partners that may act as a targeting factor. Confocal immunofluorescence (Figure 1 A) revealed that these proteins were expressed at near physiological levels, were targeted to the cytoplasmic face of melanosomes and, in the case of HTF-Rab27^WT^, rescued melanosome transport defects in Rab27a null melanocytes. These observations indicate that it is highly likely that HTF-Rab27a proteins expressed in Rab27a null melanocytes interact with a group of proteins similar to that encountered by the endogenous Rab27a in wild-type melanocytes.

HTF-Rab27a proteins were purified from these cell lines by sequential immunoprecipitations (sequential steps depicted by Western blot in Figure 1 B) and co-purified proteins were visualised by silver staining (Figure 1 C) before identification using nano-liquid chromatography mass spectrometry (as described in Material and Methods). This revealed a large set of Rab27a interacting proteins which were filtered based on the following criteria; i) presence in both HTF-Rab27a^WT^ and HTF-Rab27a^SF1/F4^ TAPs and absence from the negative non-transfected melan-*ash2* control TAP, ii) presence in previous proteomic studies of melanosomes from melanoma and retinal pigment epithelial cells [41]–[43]. This resulted in a short-list of candidate Rab27a targeting factors including Pmel-17, Glycoprotein non-metastatic protein B (GPNMB), the α-subunit of Na^+^,K^+^-ATPase isoform 1 (ATP1a1) and Annexin A2 (Anxa2). Of note, based on the candidate size, ATP1a1 (113 kDa) and Anxa2 (36 kDa) could be assigned to two of the bands observed on the silver stained gel (Figure 1 C).

### The α-subunit of Na^+^,K^+^-ATPase isoform 1 (ATP1a1) regulates melanosome transport

**Figure 2:**
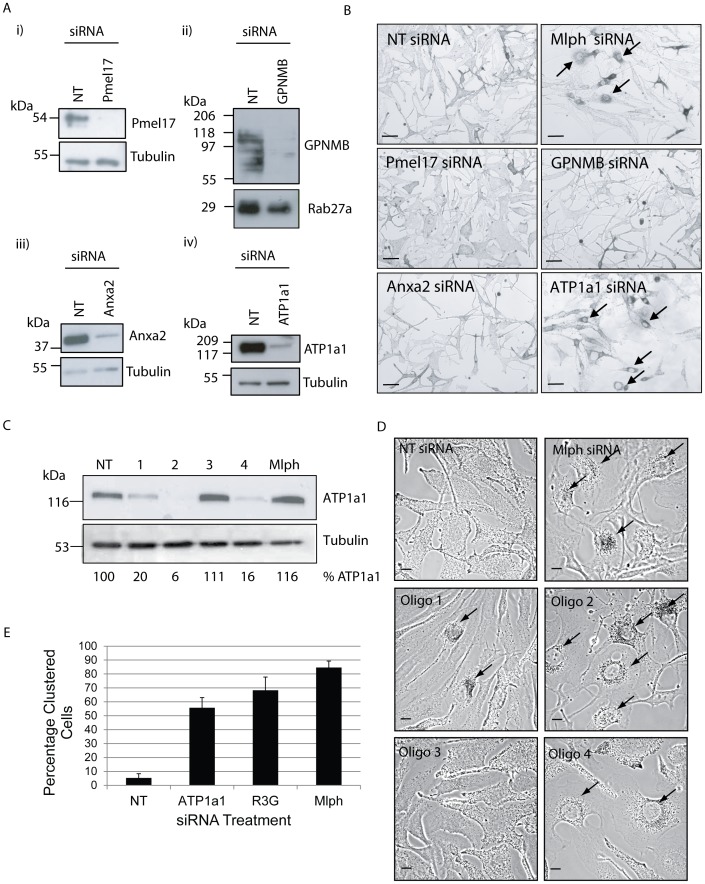
Candidate protein depletion and effects on melanosome distribution. Melan-INK4a cells were treated with siRNA pools for NT, Pmel17, GPNMB, Anxa2, ATP1a1 (A+B) or individual siRNAs for NT, ATP1a1 (1–4) or Mlph for 72 h (C+D). A) Candidate protein depletion was assessed by immunoblotting the PNS with antibodies for the candidate protein and using tubulin or Rab27a as a loading control. B) Melanosome distribution was visualised using a light microscope. Mlph siRNA depletion was used as a positive control for melanosomes clustering. Arrows indicate cells with clustered melanosomes. Scale bar represents 100 µm. C) ATP1a1 depletion was assessed by immunoblotting the PNS with antibodies to ATP1a1 and Tubulin. Depletion was quantified by measuring band intensities using ImageJ, normalised to tubulin loading control and the percentage protein quantified relative to NT siRNA control. D) Melanosome distribution was visualised using a confocal microscope. Mlph was used as a positive control for melanosomes clustering. Arrows indicate cells with clustered melanosomes. Scale bar represents 10 µm. E) The percentage of clustered cells was quantified from three independent experiments, 2 separate coverslips per condition and counting a minimum of 100 cells per coverslip. Error bars are standard deviation.

**Figure 3:**
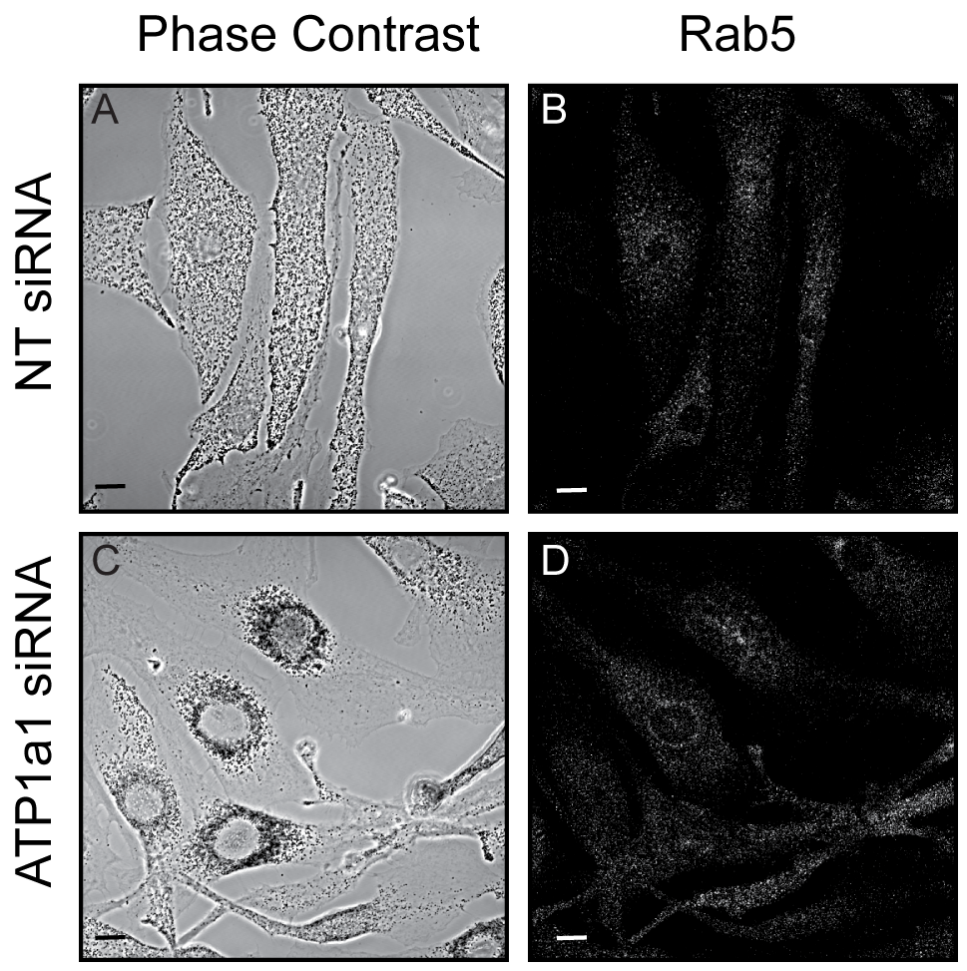
Rab5a-positive vesicle distribution is unaffected following ATP1a1 depletion. Melan-INK4a cells were treated with either NT or ATP1a1 siRNA and cultured for 72 h. Cells were fixed with PFA, permeabilised and immunolabelled with antibodies to Rab5a. Phase contrast panels show melanosome distribution (A and C), panels B and D show Rab5a staining. Scale bar represents 10 µm.

To test the role of these candidates in Rab27a targeting and function, melanosome distribution, a read-out of Rab27a function (see Introduction), was examined in wild-type melanocytes in which these proteins were individually depleted using siRNA oligonucleotide pools. This revealed that knockdown of ATP1a1, but not other candidates, redistributed melanosomes from peripheral to perinuclear accumulation suggesting that ATP1a1 functions in Rab27a-dependent melanosome transport (Figure 2 A–B). Similar results were obtained using the single siRNA pairs that comprise the ATP1a1-specific pool (Figure 2 C–D). Notably oligonucleotide pair 3 failed to deplete protein expression and similarly failed to induce melanosomal clustering whilst oligonucleotide pair 2 was 94% efficient in depleting ATP1a1 protein and caused extensive clustering, and was thus used for subsequent experiments. Quantification of the clustering phenotype found that ATP1a1 depletion resulted in 56±7% clustered cells compared to 5±3%, 68±9% and 85±5% for NT, R3G and Mlph depleted cells respectively (Figure 2 E). The specificity of the effect of ATP1a1 depletion on melanosome distribution was confirmed by the observation that the subcellular distribution of Rab5 positive endosomes was not significantly altered in knocked-down cells, which were identified by perinuclear melanosomal clustering and loss of ATP1a1 immunostaining (Figure 3). These data indicate that ATP1a1 may regulate Rab27a function in melanosome transport.

The Na^+^,K^+^-ATPase functions as an ion pump exchanging 3 Na^+^ for 2 K^+^ following hydrolysis of ATP or as a receptor for cardiotonic steroids [44]. It is a heterodimeric transmembrane complex consisting of a 10-transmembrane domain α-subunit and a single transmembrane regulatory β-subunit [45]. Four isoforms of the α-subunit have been identified [46], [47], with the α1 isoform (ATP1a1) being ubiquitously expressed [48]. To date, ATP1a1 has not been associated to any membrane trafficking event.

### The intracellular M4M5 loop of ATP1a1 interacts directly with Rab27a

**Figure 4:**
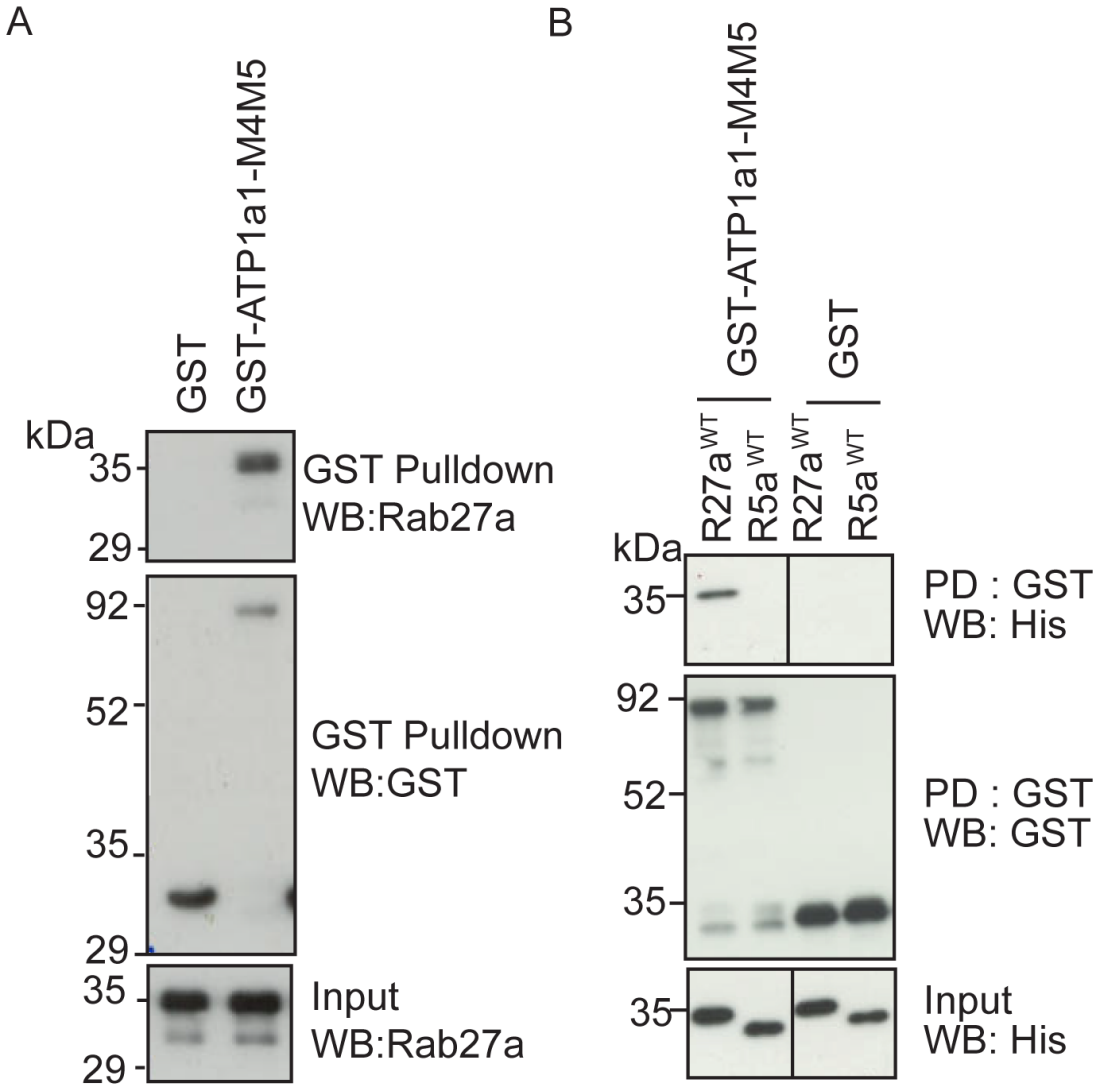
GST-ATP1a1-M4M5 interacts directly with Rab27a. GST or GST-ATP1a1-M4M5 was incubated with A) melan-INK4a cell lysates, B) his_6_-Rab27a^WT^ or his_6_-Rab5a^WT^. GST was immobilised on glutathione Sepharose. Co-precipitation was assessed by immunoblotting for Rab27a or his_6_ and GST. Blots are representative of 3 independent experiments.

Having shown an *in vivo* interaction between Rab27a and ATP1a1 by TAP and established a functional link in melanosome transport, the mechanism by which ATP1a1 regulates Rab27a-dependent melanosome transport was investigated. As a first step to address this, the nature of the interaction between Rab27a and ATP1a1 was characterised in greater detail. To do this, pull-down experiments that measure the ability of GST-tagged ATP1a1 to stably associate with either native Rab27a from melanocyte lysates, or his_6_-tagged Rab27a purified from bacteria were performed (see Materials and Methods). For these experiments the large intracellular loop of ATP1a1 that lies between trans-membrane domains 4 and 5 (henceforth ATP1a1-M4M5) was used, as previous studies have shown that this loop mediates the association with intracellular proteins [36], [49]–[53]. Consistent with the results of the TAP experiments (described above), in both instances Rab27a was precipitated by GST-ATP1a1-M4M5 but not GST alone (Figure 4 A–B). The specificity of this was further underlined by the lack of interaction between his_6_-Rab5a and GST-ATP1a1-M4M5 (Figure 4 B). These observations indicate that Rab27a and the intracellular M4M5 loop of ATP1a1 interact specifically and directly. These data, together with apparently similar effects of knockdown of either protein (Figure 2 and previous data [38]), suggest that Rab27a and ATP1a1 work together to regulate melanosome transport.

### ATP1a1 depletion reduces the level of active Rab27a in melanocytes

**Figure 5:**
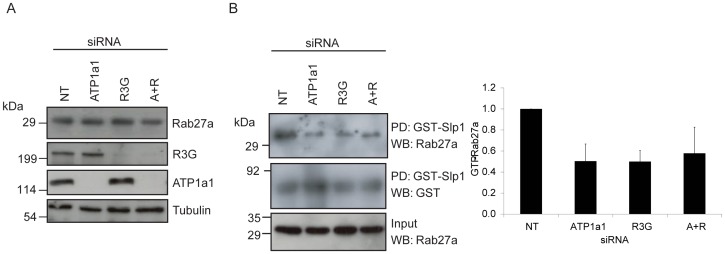
ATP1a1 depletion does not affect expression but decreases GTP-loading of Rab27a. Melan-INK4a cells were treated with siRNA oligos as indicated. A) PNS of cells transfected with the siRNA indicated were immunoblotted with antibodies indicated. B) Solubilised cell lysates were incubated with immobilised GST or GST-Slp1. Co-precipitation was assessed by immunoblotting for Rab27a and GST. The immunoblot is representative of 5 independent experiments. Depletion was quantified by measuring band intensities using ImageJ, normalised to GST-Slp1 and the protein quantified relative to NT siRNA control. Quantification of 5 independent Slp1 pulldown experiments. Error bars indicate SEM.

To further explore this idea, the effect of ATP1a1 depletion on Rab27a protein expression and activity was investigated. Western blotting of lysates from siRNA transfected melanocyte (from five independent experiments) indicated that overall Rab27a expression, relative to tubulin, was not strongly affected by ATP1a1 knockdown (densitometric measurements normalised to NT siRNA were 1.04±0.39, 1.20±0.38 and 1.01±0.6 for ATP1a1, R3G or ATP1a1+R3G depleted cells, respectively; Figure 5 A). In contrast, Rab27 effector pull-down assays, which report the level of active GTP-bound Rab27a in melanocyte lysates, revealed a 50% reduction in the level of active Rab27a in ATP1a1 depleted versus control NT transfected cells (Figure 5 C–D; data representative of 5 independent experiments). This suggests that ATP1a1 regulates the level of active Rab27a. Previous studies revealed a similar reduction in active Rab27a in melanocytes depleted of R3G [30], however Western blotting revealed that R3G expression levels are normal in ATP1a1 depleted cells and vice versa indicating that the reduction in activity did not result from reduction in R3G expression (Figure 5 A). Considering the comparable reduction in active Rab27a in cells depleted of ATP1a1 and R3G either individually or in combination, this suggests that ATP1a1 and R3G may work together to regulate the levels of active Rab27a in melanocytes.

### ATP1a1 depletion reduces Rab27a targeting to melanosomes in melanocytes

**Figure 6:**
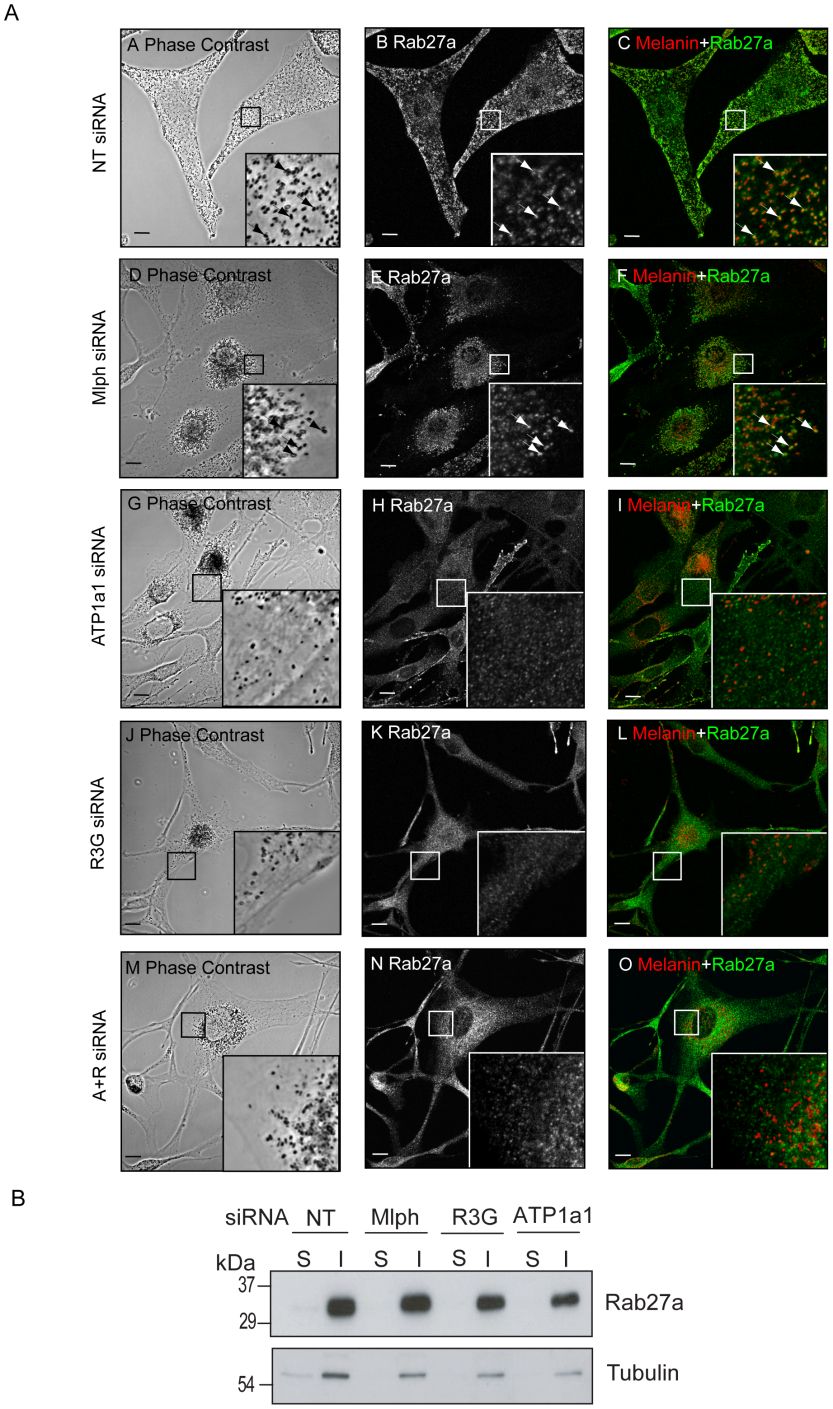
ATP1a1 depletion disrupts endogenous Rab27a targeting to melanosomes. A) Melan-INK4a cells were plated on coverslips and treated with NT (A–C), Mlph (D–F), ATP1a1 (G–I), R3G (J–L) or ATP1a1+R3G (M–O) siRNA for 72 h before repeating the treatment. After a total of 7 days, cells were fixed with PFA, permeabilised and immunolabelled with an antibody to Rab27a. Phase contrast panels show melanosome distribution (A, D, G, J, M); panels B, E, H, K, N show Rab27a localisation. In the merge panels (C, F, I, L, O) the pigment is inverted and pseudo-coloured red to aid co-localisation with the green Rab27a signal. Insets are a higher magnification of the boxed area. Arrows indicate co-localisation. Scale bar represents 10 µm. B) Melan-INK4a cells were treated with NT, Mlph, R3G or ATP1a1 siRNA and cultured for 3 days. The PNS was then separated into the insoluble (I) and soluble (S) fraction by centrifugation at 100,000×*g*. Rab27a and Tubulin partitioning were assessed by immunoblotting with the antibodies indicated.

**Figure 7:**
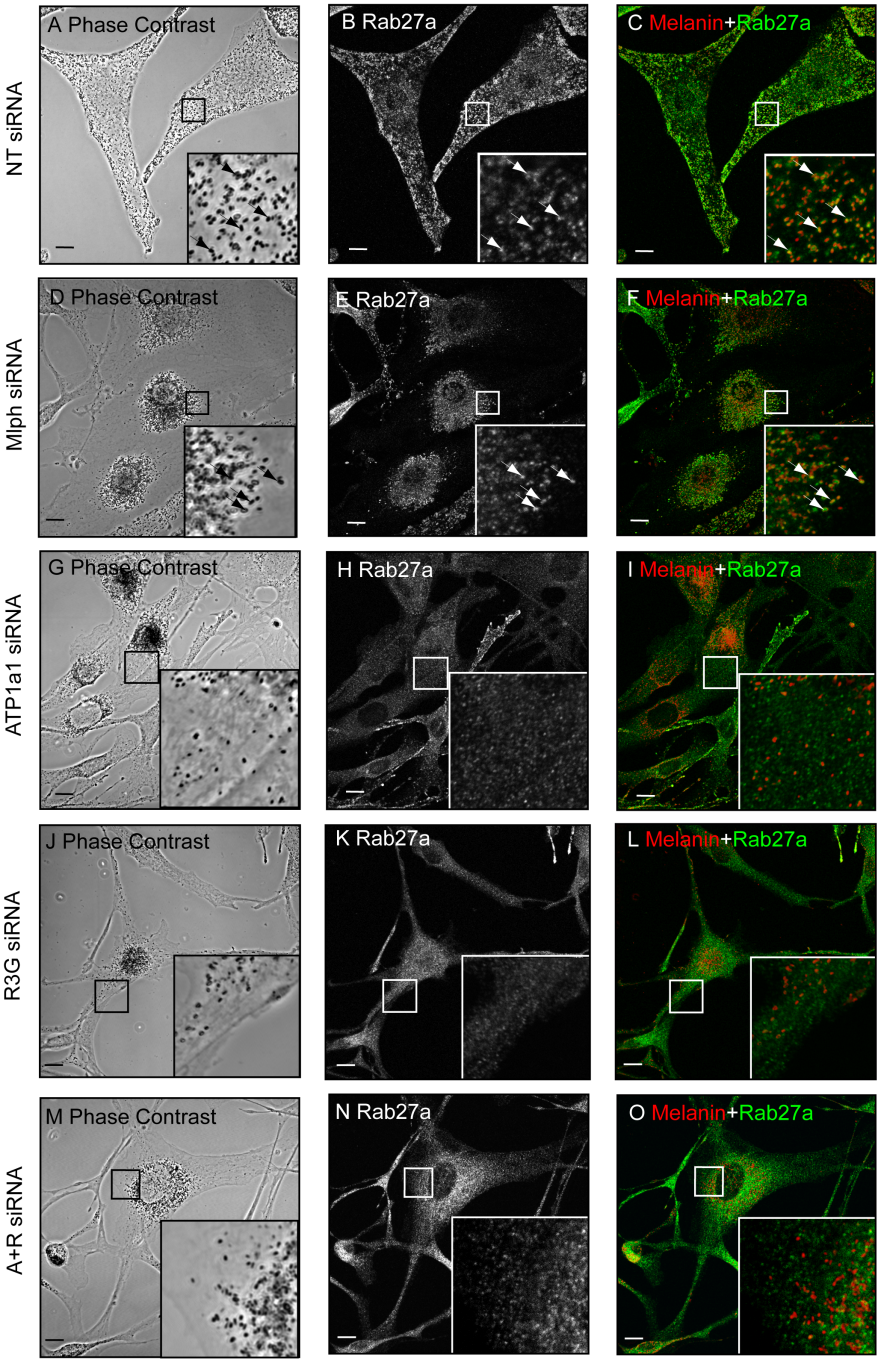
Rab38 localisation to melanosomes is unaffected following ATP1a1 depletion. Melan-INK4a cells were treated with either NT or ATP1a1 siRNA and cultured for 72 h. Cells were fixed with PFA, permeabilised and immunolabelled with antibodies to Rab38 or ATP1a1. Phase contrast panels show melanosome distribution (A, D, G, J), immunostaining is shown for Rab38 (B and E) and ATP1a1 (H and K). In the merge panels (C, F, I, L) the pigment is inverted and pseudo-coloured red to aid co-localisation with the green immunoflrescence signals. Insets are a higher magnification of the boxed area. Arrows indicate co-localisation between pigment and Rab38 (C and F). Scale bar represents 10 µm.

The above observations together with previous reports of R3G as a regulator of Rab27a targeting to melanosomes [13] raised the interesting possibility that ATP1a1 might play a similar role. To test this possibility, the subcellular distribution of endogenous Rab27a in ATP1a1-depleted wild-type melanocytes was investigated using confocal immunofluorescence microscopy (Figure 6). In control (NT) oligonucleotide transfected (Figure 6 A, panels A–C) and Mlph-depleted cells (Figure 6 A, panels D–F), the distribution of anti-Rab27a staining and melanosomes was closely correlated, indicating that Rab27a associates with melanosomes. In contrast, anti-Rab27a staining in ATP1a1 depleted cells revealed a redistribution of Rab27a from melanosomes to punctate structures that, in contrast to the perinuclear accumulated melanosomes, appeared uniformly distributed throughout the cytoplasm (Figure 6 A, panels G–I). This distribution pattern was similar to that observed for Rab27a in R3G and ATP1a1+R3G-depleted cells (Figure 6 A, panels J–O). Complementary biochemical separation of the post-nuclear supernatant of siRNA transfected melanocytes into membrane and cytosol fractions indicate that these punctate structure likely represent a population of intracellular vesicles (Figure 6 B). This is similar to findings presented here and previously [13] that Rab27a targeting to total cellular membrane is unaffected by R3G knockdown. The effect of ATP1a1 depletion on the association of Rab27a with melanosomes appears specific as Rab38 immunostaining maintains co-localisation with melanin in the perinuclear region in ATP1a1 depleted cells (Figure 7 A–F).

In order to quantify the endogenous Rab27a associated with melanosomal membranes, melanosomes were purified from siRNA treated melan-INK4a as described previously [40] then analysed by immunofluorescence microscopy. Quantification of the levels of Rab27a immunofluorescence associated with individual purified melanosomes observed significant reductions after ATP1a1 (−22±5%), R3G (−33±10%) and ATP1a1+R3G (−50±5%) siRNA depletion. The same was repeated to analyse Rab38 association and a trend towards an increase was observed following ATP1a1 (+26±8%) or R3G (+21±16%) that was only significant after dual depletion (+46±21%). Crucially, Mlph measurements saw significant reductions following ATP1a1 (−62±7%), R3G (−79±3%) and ATP1a1+R3G (−72±3%) siRNA depletion supporting the previous data showing a reduction in active Rab27a (Figure 5 C–D)

These results support a role for ATP1a1 in targeting Rab27a to melanosomes.

### A RabSF2 dependent, nucleotide independent Rab27a∶ATP1a1 interaction is essential for Rab27a targeting to melanosomes

**Figure 8:**
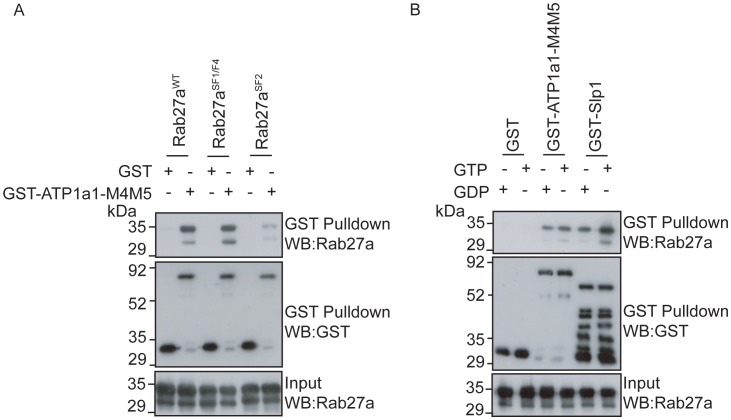
Rab27a∶ATP1a1 interaction is essential for Rab27a targeting. GST or GST-ATP1a1-M4M5 was incubated with A) his_6_-Rab27a^WT^, his_6_-Rab27a^SF1/F4^ or his_6_-Rab27a^SF2^ or B) GDP or GTPγS-loaded his_6_-Rab27a. GST-Slp1 was used as a positive control in B. GST was immobilised on glutathione Sepharose. Co-precipitation was assessed by immunoblotting for Rab27a and GST. Blots are representative of 3 independent experiments.

To characterise the significance of the direct Rab27a∶ATP1a1 interaction in Rab27a targeting to melanosomes, the ATP1a1-M4M5 pull-down assay was used to measure the ability of targeted and mis-targeted Rab27a mutants to interact with ATP1a1 *in vitro*. This revealed that while both Rab27a^WT^ and the targeted mutant (Rab27a^SF1/F4^) interacted with ATP1a1 with similar efficiency, the interaction was reduced approximately ten-fold for the non-targeted mutant (Rab27a^SF2^) (Figure 8 A). This suggests that the RabSF2 motif is necessary for Rab27a binding to the large cytoplasmic loop of ATP1a1 and subsequent targeting of Rab27a to melanosomes.

As current models indicate that Rabs initially associate with target membranes in a GDP-bound state [54], this assay was repeated using Rab27a pre-loaded with either GTPγS or GDP to determine whether the Rab27a∶ATP1a1 interaction is dependent upon the nucleotide/activation status of Rab27a. Strikingly, GST-ATP1a1-M4M5 interacted equally efficiently with either GTPγS- or GDP-loaded Rab27a (Figure 8 B). This was in marked contrast to GST-Slp1 which, as expected, interacted more strongly with GTPγS- than GDP-loaded Rab27a (Figure 8 B). As there is yet to be a consensus on whether Rabs are recruited in a GTP- or GDP-loaded state, our data would support a hypothesise that a Rab would first be recruited in the GDP-bound state and presented to the GEF for GTP-loading and subsequent retention on the membrane. Therefore the factor responsible for this would need to be able to associate with the Rab in both nucleotide bound states.

These data indicate that ATP1a1 is not a Rab27a effector and provide further support for the hypothesis that ATP1a1 regulates Rab27a targeting.

## Discussion

In this study we investigated the mechanism of Rab targeting to intracellular compartments using Rab27a targeting to melanosomes in melanocytes as a model. We present findings that strongly support an essential role for ATP1a1 in the targeting of Rab27a to melanosomes. Firstly, using tandem affinity purification we identified ATP1a1 as a Rab27a interacting protein in melanocytes. *In vitro* we then showed that the large intracellular loop of ATP1a1 (ATP1a1-M4M5) directly interacts with Rab27a. Furthermore, we showed that this interaction is Rab27a specific, independent of the Rab27a nucleotide-bound state and that the ability of Rab27a mutants to interact with ATP1a1 correlates with their ability to target to melanosomes. Crucially, we showed that siRNA depletion of ATP1a1 resulted in mis-localisation of Rab27a to non-melanosomal membrane structures, which coincided with a reduction in Rab27a GTP-loading and Mlph association with melanosomes. Based on these findings we propose a working model for Rab27a targeting in which a direct interaction between ATP1a1 and Rab27a, involving the RabSF2 motif and ATP1a1-M4M5, targets Rab27a to the melanosome, whereupon it becomes GTP-loaded by R3G. Rab27a-GTP may then interact with effectors e.g. Mlph/MyoVa, and function in melanosome transport.

While our proposed model advances our understanding of the mechanism targeting Rab27a to melanosomes, it also raises interesting questions about how ATP1a1 contributes to this process. Foremost among these is whether a melanosome associated pool of ATP1a1 functions like a receptor mediating the first contact between the melanosome membrane and Rab27a. In support of this, two previous studies reported ATP1a1 to be present in melanosomes purified from melanoma and RPE cells [41], [42]. However, in contrast to those studies and the results of our Rab27a TAP studies, we were unable to observe significant co-localisation between endogenous ATP1a1 and pigmented melanosomes using immunofluorescence microscopy (Figure 7 G–L). These observations suggest either that the melanosomal pool of ATP1a1 is below the detection limit of IF or that Rab27a must interact with ATP1a1, present in other membranes, en route to the melanosome. Consistent with the latter possibility most studies indicate that the majority of Na^+^,K^+^-ATPase is present at the plasma membrane and may be enriched in caveolae [55]. This might suggest that melanosomes, or other membranes through which Rab27a transits en route to melanosomes, must be in close proximity to the plasma membrane at the time of Rab27a recruitment. Such a mechanism would fit with current models of melanosome transport in which Rab27a, and its effectors Mlph and MyoVa, tethers melanosomes within peripheral dendrites via interaction with F-actin. Indeed, the restriction of Rab27a recruitment to occur in close proximity of the plasma membrane might provide additional regulation of melanosome transport.

A direct protein-protein interaction-type functional link between plasma membrane ATP1a1 and intracellular organelles has been shown previously. Work in rat renal proximal tubules showed that ATP1a1 and inositol 1,4,5-trisphosphate (IP3) receptor (IP3R) interact in response to ouabain, resulting in release of Ca^2+^ from ER stores independent of IP3 [56]. Moreover, this study reported a direct interaction between the intracellular N-terminus 95 amino acids of ATP1a1 and IP3R *in vitro*. These observations support the idea that ATP1a1 at the plasma membrane may regulate the function of intracellular proteins and organelles via direct protein∶protein interactions.

Another interesting question is the extent to which Na^+^, K^+^-ATPase ion pump function might regulate melanosomal Rab27a recruitment via regulation of melanosomal ion homeostasis. It is likely that the luminal ion environment would influence the conformational state of ATP1a1. This conformational state could relay across the melanosomal membrane the maturation stage of the melanosome and subsequent ability to recruit Rab27a. A precedent for ion transporters recruiting small GTPases comes from work showing recruitment of Arf6 and its GEF ARNO on to maturing endosomes is dependent on luminal pH and an interaction with the V-ATPase [57]. Alternatively, ion homeostasis could influence Rab27a targeting indirectly via regulation of R3G activity. Furthermore, previous studies have suggested that ion transporters play a role in regulating melanogenesis [58]–[60]. Therefore, the Na^+^,K^+^-ATPase may additionally contribute to melanosomal ion homeostasis and subsequent melanosome maturation.

This is the first reported incidence of a Rab protein interaction required for correct localisation involving a protein with no known Rab regulatory or effector function. Further work should unravel the precise mechanism by which ATP1a1 regulates Rab27a targeting and how this is coordinated with R3G activity.

## References

[1] Stenmark H (2009) Rab GTPases as coordinators of vesicle traffic. Nat Rev Mol Cell Biol 10: 513–525. 19603039 10.1038/nrm2728

[2] Seabra MC, Wasmeier C (2004) Controlling the location and activation of Rab GTPases. Curr Opin Cell Biol 16: 451–457. 15261679 10.1016/j.ceb.2004.06.014

[3] Booth AEG, Hume AN, Seabra MC (2012) Rab27a and melanosomes: a model to investigate the membrane targeting of Rabs. Biochem Soc Trans 40: 1383–1388. 23176485 10.1042/BST20120200

[4] Chavrier P, Gorvel JP, Stelzer E, Simons K, Gruenberg J, et al (1991) Hypervariable C-Terminal Domain of Rab Proteins Acts as a Targeting Signal. Nature 353: 769–772. 1944536 10.1038/353769a0

[5] Stenmark H, Valencia A, Martinez O, Ullrich O, Goud B, et al (1994) Distinct structural elements of rab5 define its functional specificity. EMBO J 13: 575–583. 8313902 10.1002/j.1460-2075.1994.tb06295.x PMC394846

[6] Brennwald P, Novick P (1993) Interactions of Three Domains Distinguishing the Ras-related GTP-binding Proteins Ypt1 and Sec4. Nature 362: 560–563. 8464498 10.1038/362560a0

[7] Li F, Yi L, Zhao L, Itzen A, Goody RS, et al (2014) The role of the hypervariable C-terminal domain in Rab GTPases membrane targeting. Proceedings of the National Academy of Sciences 111: 2572–2577. 10.1073/pnas.1313655111 PMC3932868 24550285

[8] Aivazian D, Serrano RL, Pfeffer S (2006) TIP47 is a Key Effector for Rab9 Localization. J Cell Biol 173: 917–926. 16769818 10.1083/jcb.200510010 PMC2063917

[9] Sivars U, Aivazian D, Pfeffer S (2005) Purification and Properties of Yip3/PRA1 as a Rab GDI Displacement Factor. Methods in Enzymology: Academic Press. pp. 348–356. 10.1016/S0076-6879(05)03030-2 16473601

[10] Sivars U, Aivazian D, Pfeffer SR (2003) Yip3 Catalyses the Dissociation of Endosomal Rab-GDI Complexes. Nature 425: 856–859. 14574414 10.1038/nature02057

[11] Dirac-Svejstrup AB, Sumizawa T, Pfeffer SR (1997) Identification of a GDI Displacement Factor that Releases Endosomal Rab GTPases from Rab-GDI. EMBO J 16: 465. 9034329 10.1093/emboj/16.3.465 PMC1169650

[12] Zhang X, He X, Fu XY, Chang Z (2006) Varp is a Rab21 Guanine Nucleotide Exchange Factor and Regulates Endosome Dynamics. J Cell Sci 119: 1053–1062. 16525121 10.1242/jcs.02810

[13] Tarafder AK, Wasmeier C, Figueiredo AC, Booth AEG, Orihara A, et al (2011) Rab27a Targeting to Melanosomes Requires Nucleotide Exchange but Not Effector Binding. Traffic 12: 1056–1066. 21554507 10.1111/j.1600-0854.2011.01216.x PMC3509405

[14] Schoebel S, Oesterlin LK, Blankenfeldt W, Goody RS, Itzen A (2009) RabGDI Displacement by DrrA from Legionella Is a Consequence of Its Guanine Nucleotide Exchange Activity. Mol Cell 36: 1060–1072. 20064470 10.1016/j.molcel.2009.11.014

[15] Cabrera M, Ungermann C (2013) Guanine Nucleotide Exchange Factors (GEFs) Have a Critical but Not Exclusive Role in Organelle Localization of Rab GTPases. Journal of Biological Chemistry 288: 28704–28712. 23979137 10.1074/jbc.M113.488213 PMC3789967

[16] Barr FA (2013) Rab GTPases and membrane identity: Causal or inconsequential? The Journal of Cell Biology 202: 191–199. 23878272 10.1083/jcb.201306010 PMC3718981

[17] Izumi T (2003) The roles of Rab27 and its effectors in the regulated secretory pathways. Cell Struct Funct 28: 465. 14745138 10.1247/csf.28.465

[18] Fukuda M (2005) Versatile Role of Rab27 in Membrane Trafficking: Focus on the Rab27 Effector Families. J Biochem 137: 9–16. 15713878 10.1093/jb/mvi002

[19] Wilson SM (2000) A Mutation in Rab27a Causes the Vesicle Transport Defects Observed in ashen Mice. Proc Natl Acad Sci USA 97: 7933. 10859366 10.1073/pnas.140212797 PMC16648

[20] Menasche G (2000) Mutations in RAB27A cause Griscelli Syndrome Associated with Haemophagocytic Syndrome. Nat Genet 25: 173. 10835631 10.1038/76024

[21] Bahadoran P, Aberdam E, Mantoux F, Busca R, Bille K, et al (2001) Rab27a: A Key to Melanosome Transport in Human Melanocytes. J Cell Biol 152: 843. 11266474 10.1083/jcb.152.4.843 PMC2195788

[22] Hume AN, Collinson LM, Rapak A, Gomes AQ, Hopkins CR, et al (2001) Rab27a Regulates the Peripheral Distribution of Melanosomes in Melanocytes. J Cell Biol 152: 795. 11266470 10.1083/jcb.152.4.795 PMC2195786

[23] Wu X, Bowers B, Roa K, Wei Q, Hammer JA 3rd (1998) Visualization of Melanosome Dynamics Within Wild-Type and Dilute Melanocytes Suggests a Paradigm for Myosin V Function In vivo. J Cell Biol 143: 1899–1918. 9864363 10.1083/jcb.143.7.1899 PMC2175227

[24] Matesic LE, Yip R, Reuss AE, Swing DA, O'Sullivan TN, et al (2001) Mutations in Mlph, Encoding a Member of the Rab Effector Family, Cause the Melanosome Transport Defects Observed in leaden Mice. Proc Natl Acad Sci USA 98: 10238–10243. 11504925 10.1073/pnas.181336698 PMC56945

[25] Wasmeier C, Hume AN, Bolasco G, Seabra MC (2008) Melanosomes at a glance. Journal of Cell Science 121: 3995–3999. 19056669 10.1242/jcs.040667

[26] Slominski A, Zmijewski MA, Pawelek J (2012) L-tyrosine and L-dihydroxyphenylalanine as hormone-like regulators of melanocyte functions. Pigment Cell & Melanoma Research 25: 14–27. 21834848 10.1111/j.1755-148X.2011.00898.x PMC3242935

[27] Slominski A, Tobin DJ, Shibahara S, Wortsman J (2004) Melanin Pigmentation in Mammalian Skin and Its Hormonal Regulation. Physiol Rev 84: 1155–1228. 15383650 10.1152/physrev.00044.2003

[28] Ali BR, Wasmeier C, Lamoreux L, Strom M, Seabra MC (2004) Multiple Regions Contribute to Membrane Targeting of Rab GTPases. J Cell Sci 117: 6401–6412. 15561774 10.1242/jcs.01542

[29] Pereira-Leal JB, Seabra MC (2000) The Mammalian Rab Family of Small GTPases: Definition of Family and Subfamily Sequence Motifs Suggests a Mechanism for Functional Specificity in the Ras Superfamily. J Mol Biol 301: 1077–1087. 10966806 10.1006/jmbi.2000.4010

[30] Figueiredo AC, Wasmeier C, Tarafder AK, Ramalho JS, Baron RA, et al (2008) Rab3GEP Is the Non-redundant Guanine Nucleotide Exchange Factor for Rab27a in Melanocytes. J Biol Chem 283: 23209–23216. 18559336 10.1074/jbc.M804134200 PMC2516999

[31] Blümer J, Rey J, Dehmelt L, Mazel T, Wu Y-W, et al (2013) RabGEFs are a major determinant for specific Rab membrane targeting. The Journal of Cell Biology 200: 287–300. 23382462 10.1083/jcb.201209113 PMC3563681

[32] Gerondopoulos A, Langemeyer L, Liang J-R, Linford A, Barr Francis A (2012) BLOC-3 Mutated in Hermansky-Pudlak Syndrome Is a Rab32/38 Guanine Nucleotide Exchange Factor. Current Biology 22: 2135–2139. 23084991 10.1016/j.cub.2012.09.020 PMC3502862

[33] Wasmeier C, Romao M, Plowright L, Bennett DC, Raposo G, et al (2006) Rab38 and Rab32 Control Post-Golgi Trafficking of Melanogenic Enzymes. J Cell Biol 175: 271–281. 17043139 10.1083/jcb.200606050 PMC2064568

[34] Hume AN, Tarafder AK, Ramalho JS, Sviderskaya EV, Seabra MC (2006) A Coiled-Coil Domain of Melanophilin Is Essential for Myosin Va Recruitment and Melanosome Transport in Melanocytes. Mol Biol Cell 17: 4720–4735. 16914517 10.1091/mbc.E06-05-0457 PMC1635380

[35] Sviderskaya EV, Hill SP, Evans-Whipp TJ, Chin L, Orlow SJ, et al (2002) p16Ink4a in Melanocyte Senescence and Differentiation. J Natl Cancer Inst 94: 446–454. 11904317 10.1093/jnci/94.6.446

[36] Alves DS, Farr GA, Seo-Mayer P, Caplan MJ (2010) AS160 Associates with the Na+,K+-ATPase and Mediates the Adenosine Monophosphate-stimulated Protein Kinase-dependent Regulation of Sodium Pump Surface Expression. Mol Biol Cell 21: 4400–4408. 20943949 10.1091/mbc.E10-06-0507 PMC3002392

[37] Leung KF, Baron R, Seabra MC (2006) Thematic Review Series: Lipid Posttranslational Modifications. Geranylgeranylation of Rab GTPases. J Lipid Res 47: 467–475. 16401880 10.1194/jlr.R500017-JLR200

[38] Hume AN, Ushakov DS, Tarafder AK, Ferenczi MA, Seabra MC (2007) Rab27a and MyoVa are the Primary Mlph Interactors Regulating Melanosome Transport in Melanocytes. J Cell Sci 120: 3111–3122. 17698919 10.1242/jcs.010207

[39] Strom M, Hume AN, Tarafder AK, Barkagianni E, Seabra MC (2002) A Family of Rab27-Binding Proteins. Melanophilin Links Rab27a and Myosin Va Function in Melanosome Transport. J Biol Chem 277: 25423. 11980908 10.1074/jbc.M202574200

[40] Chabrillat ML, Wilhelm C, Wasmeier C, Sviderskaya EV, Louvard D, et al (2005) Rab8 Regulates the Actin-based Movement of Melanosomes. Mol Biol Cell 16: 1640–1650. 15673612 10.1091/mbc.E04-09-0770 PMC1073648

[41] Chi A, Valencia JC, Hu Z-Z, Watabe H, Yamaguchi H, et al (2006) Proteomic and Bioinformatic Characterization of the Biogenesis and Function of Melanosomes. J Proteome Res 5: 3135–3144. 17081065 10.1021/pr060363j

[42] Azarian SM, McLeod I, Lillo C, Gibbs D, Yates JR, et al (2006) Proteomic Analysis of Mature Melanosomes from the Retinal Pigmented Epithelium. J Proteome Res 5: 521–529. 16512666 10.1021/pr0502323

[43] Basrur V, Yang F, Kushimoto T, Higashimoto Y, Yasumoto K-i, et al (2002) Proteomic Analysis of Early Melanosomes: Identification of Novel Melanosomal Proteins. J Proteome Res 2: 69–79. 10.1021/pr025562r 12643545

[44] Xie Z, Askari A (2002) Na+/K+-ATPase as a signal transducer. Eur J Biochem 269: 2434–2439. 12027880 10.1046/j.1432-1033.2002.02910.x

[45] Kaplan JH (2002) Biochemistry of Na,K-ATPase. Annu Rev Biochem 71: 511–535. 12045105 10.1146/annurev.biochem.71.102201.141218

[46] Shull GE, Lingrel JB (1986) Molecular Cloning of Three Distinct Forms of the Na^+^,K^+^-ATPase alpha-Subunit From Rat Brain. Biochemistry 261: 16788–16791. 10.1021/bi00373a001 3028470

[47] Shamraj OI, Lingrel JB (1994) A putative fourth Na+,K(+)-ATPase alpha-subunit gene is expressed in testis. Proc Natl Acad Sci USA 91: 12952–12956. 7809153 10.1073/pnas.91.26.12952 PMC45558

[48] Young RM, Lingrel JB (1987) Tissue Distribution of mRNAs Encoding the alpha isoforms and beta subunit of rat Na+,K+-ATPase. Biochem Biophys Res Commun 145: 52–58. 3036133 10.1016/0006-291x(87)91286-1

[49] Kim M, Jung J, Park C-S, Lee K (2002) Identification of the Cofilin-binding Sites in the Large Cytoplasmic Domain of Na,K-ATPase. Biochimie 84: 1021–1029. 12504282 10.1016/s0300-9084(02)00004-4

[50] Done SC, Leibiger IB, Efendiev R, Katz AI, Leibiger B, et al (2002) Tyrosine 537 within the Na+,K+-ATPase alpha-Subunit Is Essential for AP-2 Binding and Clathrin-dependent Endocytosis. J Biol Chem 277: 17108–17111. 11859087 10.1074/jbc.M201326200

[51] Devarajan P, Scaramuzzino DA, Morrow JS (1994) Ankyrin Binds to Two Distinct Cytoplasmic Domains of Na,K-ATPase alpha Subunit. Proc Natl Acad Sci USA 91: 2965–2969. 8159688 10.1073/pnas.91.8.2965 PMC43495

[52] Tian J, Cai T, Yuan Z, Wang H, Liu L, et al (2006) Binding of Src to Na+/K+-ATPase Forms a Functional Signaling Complex. Mol Biol Cell 17: 317–326. 16267270 10.1091/mbc.E05-08-0735 PMC1345669

[53] Yuan Z, Cai T, Tian J, Ivanov AV, Giovannucci DR, et al (2005) Na/K-ATPase Tethers Phospholipase C and IP3 Receptor into a Calcium-regulatory Complex. Mol Biol Cell 16: 4034–4045. 15975899 10.1091/mbc.E05-04-0295 PMC1196317

[54] Ullrich O, Horiuchi H, Bucci C, Zerial M (1994) Membrane Association of Rab5 Mediated by GDP-dissociation Inhibitor and Accompanied by GDP/GTP Exchange. Nature 368: 157–160. 8139660 10.1038/368157a0

[55] Liu L, Mohammadi K, Aynafshar B, Wang H, Li D, et al (2003) Role of caveolae in signal-transducing function of cardiac Na+/K+-ATPase. American Journal of Physiology - Cell Physiology 284: C1550–C1560. 12606314 10.1152/ajpcell.00555.2002

[56] Miyakawa-Naito A, Uhlen P, Lal M, Aizman O, Mikoshiba K, et al (2003) Cell Signaling Microdomain with Na,K-ATPase and Inositol 1,4,5-Trisphosphate Receptor Generates Calcium Oscillations. J Biol Chem 278: 50355–50361. 12947118 10.1074/jbc.M305378200

[57] Hurtado-Lorenzo A, Skinner M, Annan JE, Futai M, Sun-Wada G-H, et al (2006) V-ATPase Interacts with ARNO and Arf6 in Early Endosomes and Regulates the Protein Degradative Pathway. Nat Cell Biol 8: 124–136. 16415858 10.1038/ncb1348

[58] Lamason RL, Mohideen M-APK, Mest JR, Wong AC, Norton HL, et al (2005) SLC24A5, a Putative Cation Exchanger, Affects Pigmentation in Zebrafish and Humans. Science 310: 1782–1786. 16357253 10.1126/science.1116238

[59] Ginger RS, Askew SE, Ogborne RM, Wilson S, Ferdinando D, et al (2008) SLC24A5 Encodes a trans-Golgi Network Protein with Potassium-dependent Sodium-Calcium Exchange Activity That Regulates Human Epidermal Melanogenesis. J Biol Chem 283: 5486–5495. 18166528 10.1074/jbc.M707521200

[60] Smith DR, Spaulding DT, Glenn HM, Fuller BB (2004) The Relationship Between Na+/H+ Exchanger Expression and Tyrosinase Activity in Human Melanocytes. Exp Cell Res 298: 521–534. 15265699 10.1016/j.yexcr.2004.04.033

