# Correction: A Role for Na+,K+-ATPase α1 in Regulating Rab27a Localisation on Melanosomes

**DOI:** 10.1371/journal.pone.0148719

**Published:** 2016-02-03

**Authors:** Antonia E. G. Booth, Abul K. Tarafder, Alistair N. Hume, Chiara Recchi, Miguel C. Seabra

Fig 7, “Rab38 localisation to melanosomes is unaffected following ATP1a1 depletion,” is an inadvertent duplication of panel A in Figure 6. Please view Fig 7 here.

**Fig 7:**
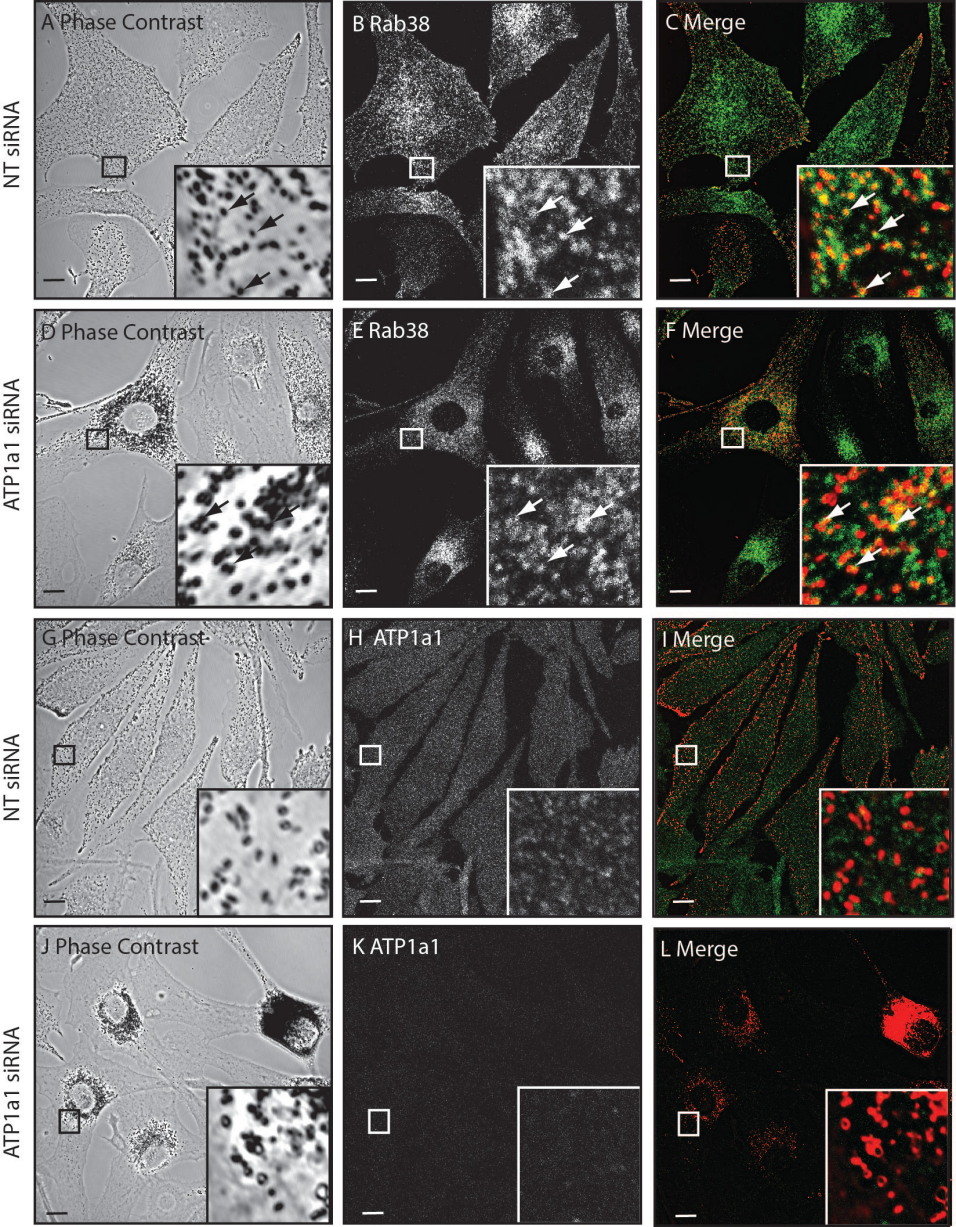
Rab38 localisation to melanosomes is unaffected following ATP1a1 depletion. Melan-INK4a cells were treated with either NT or ATP1a1 siRNA and cultured for 72 h. Cells were fixed with PFA, permeabilised and immunolabelled with antibodies to Rab38 or ATP1a1. Phase contrast panels show melanosome distribution (A, D, G, J), immunostaining is shown for Rab38 (B and E) and ATP1a1 (H and K). In the merge panels (C, F, I, L) the pigment is inverted and pseudo-coloured red to aid co-localisation with the green immunoflrescence signals. Insets are a higher magnification of the boxed area. Arrows indicate co-localisation between pigment and Rab38 (C and F). Scale bar represents 10 μm.
